# Immunohistochemical characterization and potential prognostic relevance of dopamine signaling in canine pulmonary adenocarcinoma

**DOI:** 10.3389/fvets.2025.1552345

**Published:** 2025-09-03

**Authors:** Alana R. Kuzmik, Davis M. Seelig, Luke H. Hoeppner, Aaron Rendahl, Hannah Able, Amber Wolf-Ringwall, Jessica Lawrence

**Affiliations:** ^1^Department of Veterinary Clinical Sciences, University of Minnesota, St. Paul, MN, United States; ^2^Masonic Cancer Center, University of Minnesota, Minneapolis, MN, United States; ^3^The Hormel Institute, University of Minnesota, Austin, MN, United States; ^4^Department of Surgical and Radiological Sciences, University of California, Davis, Davis, CA, United States; ^5^Department of Radiation Oncology, Medical School, University of Minnesota, Minneapolis, MN, United States

**Keywords:** cancer, pulmonary adenocarcinoma, EGFR, VEGFR2, dopamine signaling

## Abstract

**Introduction:**

Dopamine signaling contributes to tumor progression in human lung adenocarcinoma. Isoforms of dopamine-and-cAMP-regulated phosphoprotein Mr. 32,000 (DARPP-32) are overexpressed in human lung adenocarcinoma and are associated with prognosis and development of treatment resistance. Despite similarities to human lung adenocarcinoma, dopamine signaling has not yet been evaluated in canine pulmonary adenocarcinoma. The objective of this study was to characterize immunohistochemical expression of DARPP-32 isoforms in canine pulmonary adenocarcinoma and assess associations with clinical variables, markers of proliferation and angiogenesis, and outcome.

**Methods:**

Immunohistochemistry for DARPP-32 isoforms DARPP_N_ and DARPP_C_, EGFR, D2R, and VEGFR2 was used to assess 46 canine adenocarcinomas. The percentage of tumor cells positive for target protein expression and staining intensity of DARPP_C_, DARPP_N_, and EGFR were quantified using a modified immunohistochemical scoring scheme. Intratumoral vascular endothelial D2R and VEGFR2 expression were quantified based on the number of positive vessels.

**Results:**

DARPP_N_ or DARPP_C_ was expressed in 40 (87%) adenocarcinomas; 54% of tumors expressed both isoforms. DARPP_N_ was positively correlated with tumor volume, VEGFR2 expression and mitotic count. DARPP_C_ was positively correlated with EGFR expression. VEGFR2 expression was positively correlated to EGFR expression and mitotic count. When stratified by the median, survival was shorter with increased tumor volume (*p* = 0.003) and greater intratumoral VEGFR2 expression (*p* = 0.042). Dogs with DARPP_N_ (*p* = 0.059) or DARPP_C_ (*p* = 0.120) expression greater than the median had shorter survival than those with lower expression.

**Conclusion:**

Collectively, data support further investigation of DARPP-32 protein signaling in canine lung adenocarcinoma.

## 1 Introduction

Lung cancer is the leading cause of cancer and cancer death world-wide in humans (1–5). Non-small cell lung cancer (NSCLC) is the most common form of lung cancer, accounting for 85–90% of all cases (1, 3, 5). Lung adenocarcinoma has become the most prevalent histology in the past 20 years, representing 40% of all lung cancers (6). Importantly, lung adenocarcinoma is currently the most common lung cancer histology among never-smokers (3, 6). It also occurs more commonly in women compared to men, is more likely to occur in younger people than other lung cancers and is more likely to present as advanced disease (3, 6).

Pet dogs may be a naturally-occurring model of human never-smoker lung adenocarcinoma (7–9). As in humans, the majority of canine primary lung tumors are carcinomas, of which adenocarcinoma is the most common histologic subtype (10, 11). In both species, key prognostic factors such as tumor size, histologic grade and presence of lymph node metastasis are identical (8–15). Some shared molecular signatures have been documented, including overexpression of the tyrosine kinase receptor epidermal growth factor receptor (EGFR) (8). Other molecular factors such as HER2/ErbB2 and PDGFRα overexpression have also been documented (14). To date, no convincing predisposing factors for lung adenocarcinoma, such as exposure to environmental tobacco smoke, have been identified in the dog (16, 17). Surgical resection is the mainstay of treatment for early-stage disease in both humans and dogs (3, 5, 10, 11, 13, 18). Incorporation of targeted therapies such as inhibitors of epidermal growth factor receptor (EGFR) or vascular endothelial growth factor (VEGF) signaling have improved outcomes in human lung adenocarcinoma harboring sensitizing mutations (19, 20). However, resistance to such therapies typically develops and limits long-term success (3, 21–24). Indeed, although the 5-year survival rate of lung adenocarcinoma has improved over the last decade, it remains below 20–30% (3, 4). In two canine studies, EGFR and VEGFR2 were shown to be expressed in canine lung carcinomas, but their prognostic significance is unclear (8, 14). To date, there is little evidence to support efficacy of chemotherapy or targeted therapies in dogs with lung adenocarcinoma in the postoperative or non-surgical setting (11–13, 18, 25). Given increases in lung adenocarcinoma in never-smokers combined with challenges associated with effective control of advanced disease, there is strong rationale to further evaluate the dog as a parallel population to humans for emerging comparative lung cancer efforts to define new therapeutic targets.

Dopamine signaling has recently been shown to play a pivotal role in the progression of NSCLC (1, 2, 24, 26). Dopamine D2 receptor (D_2_R) agonists have been shown to inhibit lung tumor growth in part by reducing tumor angiogenesis (26). Specifically, D_2_R agonists have been shown to abrogate vascular endothelial growth factor receptor 2 (VEGFR2)-mediated signaling by facilitating endocytosis of VEGFR2 (26).

Dopamine and cyclic adenosine monophosphate-regulated phosphoprotein, Mr. 32,000 (DARPP-32) is an effector protein downstream of D_2_R that plays an important role in dopaminergic neurotransmission (1, 2, 27). Aberrant overexpression of DARPP-32 and its transcriptional splice variant, an N-terminally truncated isoform termed t-DARPP, have been associated with adenocarcinomas of the lung, stomach, esophagus, colon, and prostate (27–30). Both isoforms have been implicated in key steps of oncogenesis including cell proliferation, survival, invasion and angiogenesis (1, 2, 28–30). Specifically, DARPP-32 and t-DARPP have been associated with activating EGFR signaling and contributing to therapeutic resistance (2, 31, 32). Importantly, DARPP-32 and t-DARPP-mediated signaling in NSCLC have been shown to promote cell survival and migration, and increasing t-DARPP was associated with increased tumor size and poorer prognosis (1). Additionally, one study suggested that DARPP-32 may play a direct role in angiogenesis by increasing mRNA and protein levels of the pro-angiogenic molecule angiopoietin 2 through activation of STAT3 (33).

Dopaminergic signaling in canine pulmonary adenocarcinoma has not been assessed to date. Therefore, the primary objectives of this study were to characterize the immunohistochemical expression of DARPP-32 isoforms in canine lung adenocarcinoma, and to evaluate associations with clinical variables, EGFR and VEGFR2 immunohistochemical expression, and outcome. The hypothesis was that increased DARPP-32 isoform expression would be associated with increased EGFR and VEGFR2 expression, advanced disease, and decreased survival after surgery compared to dogs with low DARPP-32 expression.

## 2 Materials and methods

### 2.1 Case selection

Our group previously reported second opinion histopathologic characteristics, prognostic variables and outcome for dogs with primary lung tumors following CT staging and surgical removal, with or without adjuvant systemic therapy between January 2007 and November 2018 (12). Dogs with primary pulmonary adenocarcinoma were included in this study if there was sufficient tissue from retrieved paraffin-embedded tissue blocks available. Briefly, all dogs had preoperative CT scans for staging and surgical planning and underwent surgical excision of the mass with a histopathological diagnosis of primary lung neoplasia. Margin status postoperatively was classified as complete if reported margins were greater than or equal to 5 mm or if the pathologist interpreted it as complete without measurements, incomplete if reported margins were less than 1 mm or if the pathologist interpreted margins as incomplete, or narrow if the reported margins were between 1 and 5 mm or the pathologist interpreted margins as narrow. All formalin-fixed paraffin-embedded tissue blocks that had undergone second-opinion histopathology by a single board-certified veterinary pathologist in a prior study and were confirmed to be pulmonary adenocarcinoma (12) were retrieved for immunohistochemistry (IHC) staining. Institutional Animal Care and Use Committee approval was not required because tissue samples were acquired as a by-product of routine, standard-of-care treatment. Written informed consent was obtained by all owners for routine care and acquisition of tumor samples. Clinical signalment, tumor volume, metastatic status, adjuvant treatment, and survival data were retrieved for included dogs (12). Dogs were excluded if more than one pulmonary adenocarcinoma was excised at the same surgical event. If two tumors occurred at separate timepoints, only the first tumor was included for IHC. Histopathologic Variables and Immunohistochemistry (IHC).

Tumor volume and mitotic count were previously identified as prognostic for survival for 56 dogs with canine pulmonary adenocarcinoma, which included this cohort of dogs (12); these data were therefore extracted for associations with the target proteins. All histopathologic variables were manually determined by a single pathologist prior to IHC staining and analysis. To evaluate DARPP-32 isoforms, EGFR and VEGFR expression, 4 μm formalin-fixed, paraffin-embedded tissue sections from dogs with adenocarcinomas (12) were deparaffinized and rehydrated, followed by antigen retrieval using a high pH EDTA solution. After quenching endogenous peroxidase, IHC was performed using primary antibodies against D2R (Santa Cruz Biotechnology, sc-5303, 1:400), the N-terminal of DARPP-32 (DARPP_N_; Abcam, ab40801, 1:2000), the C-terminal of DARPP-32 (DARPP_C_; Santa Cruz Biotechnology, sc-398360, 1:100), EGFR (ThermoFisher MA5-13269, 1:50) and VEGFR2 (Cell Signaling 9,698, 1:1600). The DARPP_N_ antibody exclusively recognizes the full-length DARPP-32 molecule. The DARPP_C_ antibody recognizes both DARPP-32 and the N-terminally truncated isoform t-DARPP. An antibody against t-DARPP alone is not available; therefore, it can be indirectly assessed through differential IHC using DARPP_C_ and DARPP_N_ (1, 27). Primary antibodies were substituted with IgG for negative control slides. Antibodies against D2R, DARPP_N_ and DARPP_C_ were internally validated for use in the dog using canine brain tissue as a positive control. VEGFR2 was validated internally using canine splenic hemangiosarcoma and normal canine lung tissues. Antibodies against EGFR in dogs were previously validated and characterized in the literature (8, 14), and canine mammary tumors known to be positive for EGFR were used as positive controls.

A board-certified veterinary pathologist (DS) and medical oncology resident (AK), blinded to the outcome of each dog, evaluated all IHC slides. Computer-aided image analysis was not utilized. Immunostaining of DARPP_N_ and DARPP_C_ was recorded based on visual assessment of staining intensity on a 4-point scale, were 0 indicated absence of immunostaining, 1 indicated mild positivity, 2 indicated moderate positivity, and 3 indicated intensely positive immunostaining. The percentage of tumor cells fitting each intensity score category was semi-quantitatively assigned in increments of 5%. Subsequently, an immune reactivity (IR) score was calculated by multiplying staining intensity by percentage of positive tumor cells, consistent with prior literature (1, 24, 27).

Immunostaining of D2R and VEGFR2 was expected on the intratumoral vascular endothelium and not uniformly throughout the tumor tissue. Therefore, immunostaining was scored as 0 (no positive intratumoral vessels seen), 1 (1–5 positive vessels), 2 (6–10 positive vessels), 3 (11–15 positive vessels), or 4 (15 or greater positive vessels). Tumors were considered positive for EGFR expression if at least 10% of tumor cells were immunolabeled, to match previous methodology (8).

### 2.2 Statistical analysis

Statistical analysis was performed by a single biostatistician (AR) using R version 4.4.1 software (34). For categorical variables, differences were evaluated using Fisher’s exact test. To evaluate associations with binary variables, a Wilcoxon rank-sum test was used. For correlations between protein immunoreactivity scores, tumor volume and mitotic count, Spearman’s correlation was used. Correlations were defined as weak (*r*_s_ 0.20–0.39), moderate (*r*_s_ 0.40–0.69), strong (*r*_s_ 0.70–0.89), and very strong (*r*_s_ ≥ 0.90). For visualization and estimation of effect size, groups were divided by the median value. Survival time was calculated from the date of surgery. Dogs were censored if they were alive at the time of last follow-up. The median survival time (MST) was estimated with Kaplan–Meier analysis and reported as median values with 95% confidence intervals (CIs). Variable associations with survival were calculated using the log-rank test for categorical variables and O’Brien’s non-parametric test for continuous variables. O’Brien’s test was selected because it is a time-to-event (survival) analysis that allows for numeric explanatory variables, rather than binary variables (log-rank) with small datasets (35). Statistical significance was set for *p*-values <0.05.

## 3 Results

### 3.1 Clinicopathological characteristics, treatment, and outcome

Forty-eight adenocarcinomas from 46 dogs had sufficient tissue available for immunohistochemical evaluation. One dog with two concurrent pulmonary adenocarcinomas was excluded. One dog had two adenocarcinomas surgically excised at different timepoints; for this analysis, only the first tumor was included. Therefore, 46 tumors from 46 dogs were evaluable for statistical analysis.

**Figure 1:**
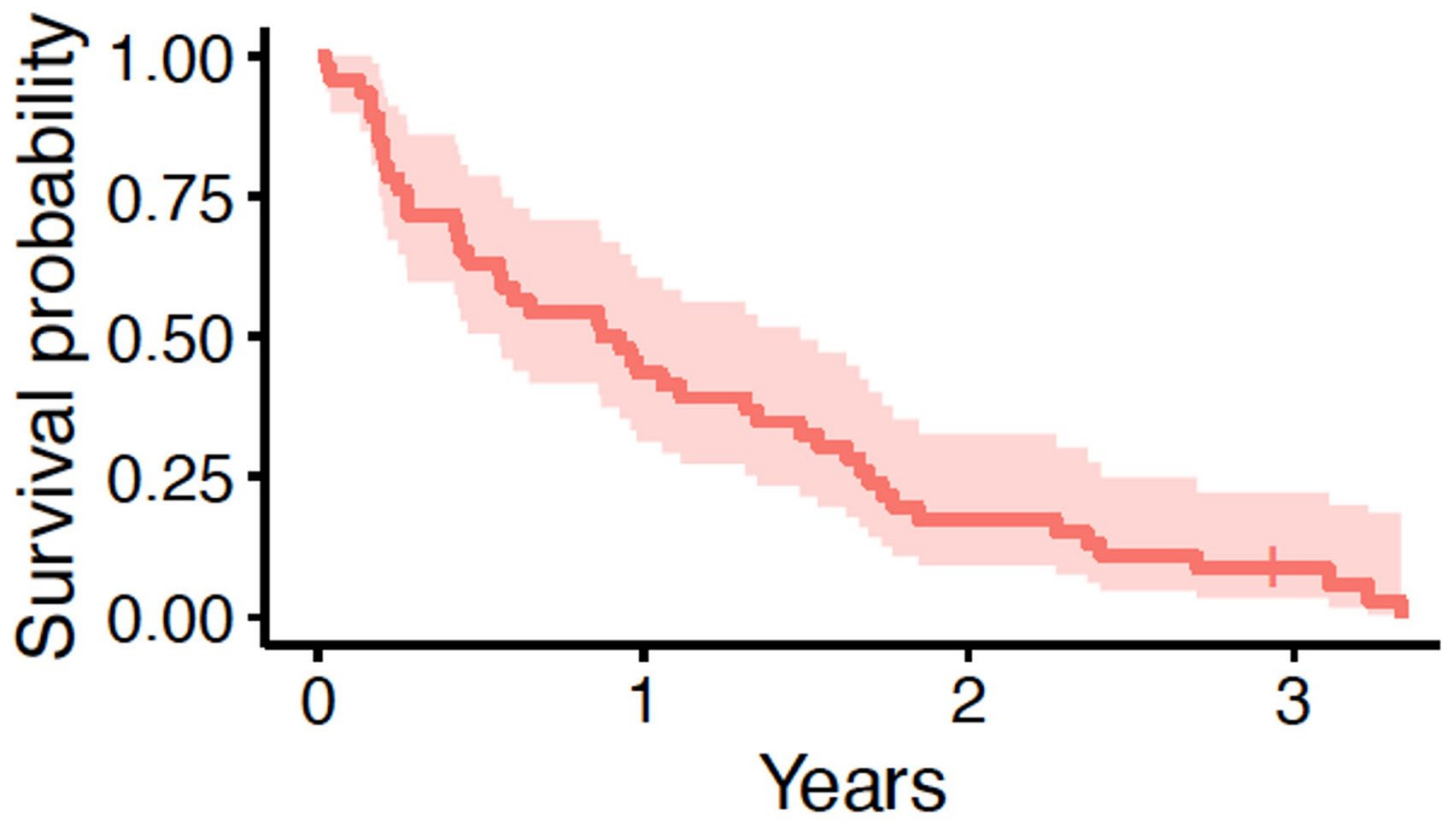
Overall survival in 46 dogs with primary lung adenocarcinoma following surgery. The tick mark represents one dog censored from analysis that was free of pulmonary neoplasia at the time of last follow-up. The median survival time was 328 days (95% CI: 204–541 days).

In this cohort of 46 dogs, the median age at the time of surgery was 11 years (range, 5.9 to 15 years) and the median body weight was 21.05 kg (range, 3.3 to 57.6 kg). Additional clinical characteristics are shown in Table 1. Tumor volumes ranged from 0.06 to 238 cm^3^, with a median tumor volume of 29.5 cm^3^. Similarly, the median mitotic count for all tumors was 9, with a wide range from 1 to 60. The majority (*N* = 34, 73.9%) of tumors had local invasion into adjacent tissues. Eight dogs received adjuvant systemic therapy following surgery; four of these dogs had complete margins, two had incomplete margins, and two dogs had narrow margins. None of the five dogs with lymph node or intrapulmonary metastasis received adjuvant therapy. Systemic therapy was prescribed at the discretion of the attending oncologist and was not standardized. Drugs prescribed included vinorelbine, carboplatin, doxorubicin, 5-fluorouracil, chlorambucil, cyclophosphamide, and toceranib. The MST for all 46 dogs was 328 days (95% CI: 204–541 days) with a range from 9 to 1,216 days (Figure 1). One dog was still alive and censored at 1072 days. Death in the majority of dogs (*N* = 34; 74%) was due to pulmonary neoplasia. Cause of death was possibly due to disease other than pulmonary adenocarcinoma in 11 dogs; however, only one dog had necropsy findings to confirm death was due to metastatic sarcoma with no evidence of pulmonary adenocarcinoma.

**Table 1:** Clinical characteristics of 46 dogs included for immunohistochemical evaluation of a primary pulmonary adenocarcinoma.

Characteristic	Number of dogs	Percentage (*N* = 46)
Sex		
Female spayed	25	54%
Male neutered	21	46%
Breed		(approximate)
Labrador Retriever	7	15%
Mixed breed	3	6%
Rat Terrier	2	4.5%
Cocker Spaniel	2	4.5%
Miniature Poodle	2	4.5%
German Shorthaired Pointer	2	4.5%
Weimaraner	2	4.5%
Jack Russell Terrier	2	4.5%
Golden Retriever	2	4.5%
American Staffordshire Terrier	2	4.5%
Other^a^	20	43%
Presence of clinical signs at diagnosis	33	71.7%
Surgical margin status		
Complete	25	54.3%
Narrow	6	13.0%
Incomplete	14	30.4%
Not reported	1	2.2%
Presence of tumor invasion	34	73.9%
Presence of intrathoracic metastasis	5	10.9%
Adjuvant postoperative systemic therapy	8	17.4%

a One dog in a variety of individual breeds.

**Figure 2:**
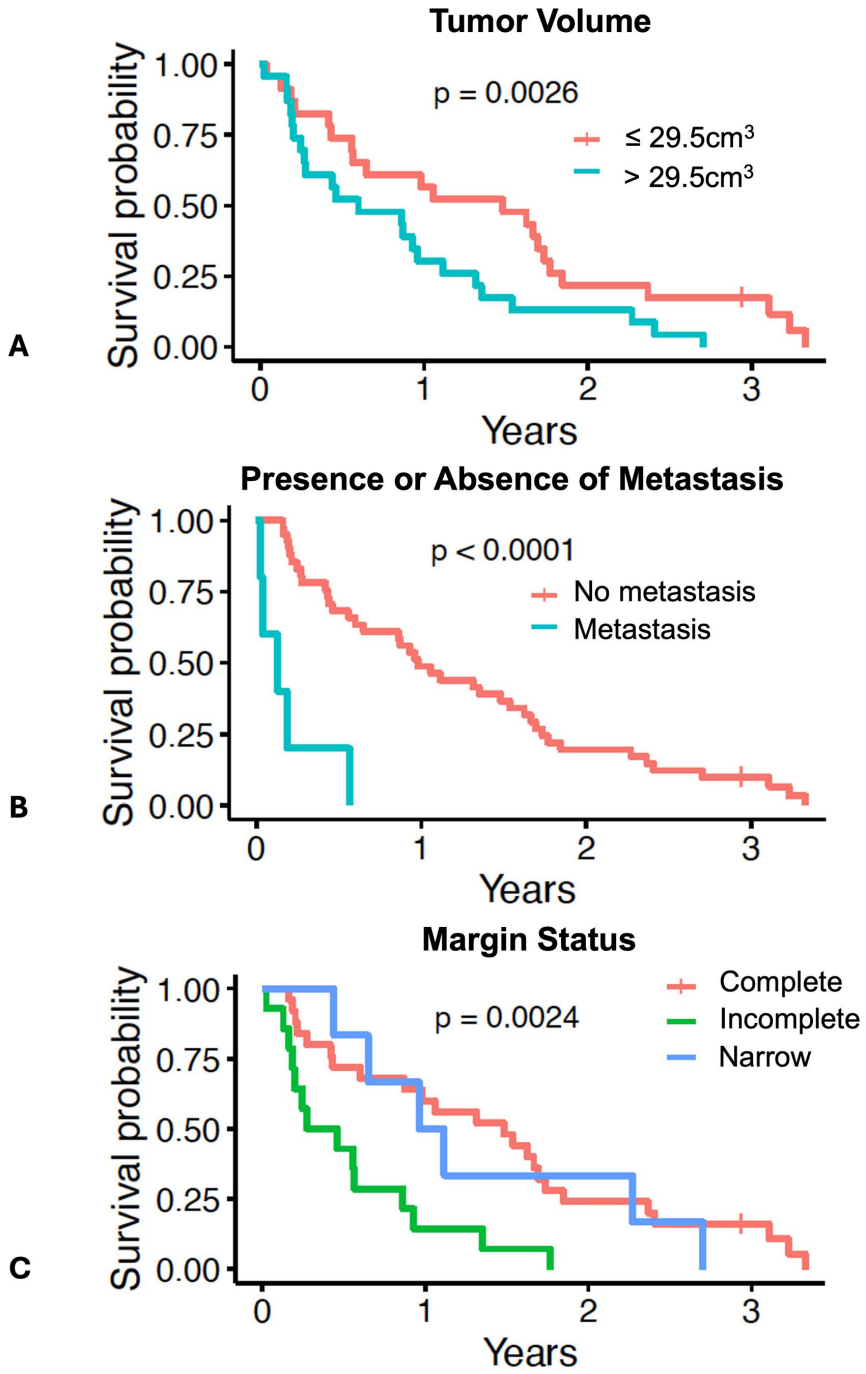
Survival in 46 dogs with primary lung adenocarcinoma following surgery, stratified by: **(A)** dogs above or below the median tumor volume of 29.5 cm^3^; **(B)** dogs with presence (*N* = 5) or absence of lymph node or pulmonary metastasis (*N* = 41); **(C)** dogs with complete margins (*N* = 25), narrow margins (*N* = 6), or incomplete margins (*N* = 14). The tick mark represents one dog censored from analysis that was free of pulmonary neoplasia at the time of last follow-up. Differences in outcome were evaluated by log-rank test for categorical variables and O’Brien’s non-parametric test for numeric variables.

In the larger dataset of dogs (*N* = 56) with pulmonary adenocarcinoma from which this cohort was drawn, tumor volume, mitotic count, and completeness of excision was prognostic for outcome, while tumor invasion into adjacent structures was associated with tumor volume (12). In this cohort of 46 dogs, when stratified by the median tumor volume, dogs with tumor volumes greater than the median (29.5 cm^3^) had worse survival than dogs with tumor volumes less than the median (Figure 2A and Table 2, *p* = 0.0026). Dogs with metastasis (*N* = 5) had shorter survival compared to dogs without metastasis (*N* = 41) (Figure 2B and Table 2, *p* < 0.0001). The median tumor volume for the dogs with metastasis was 22.8 cm^3^ (range 10.2–165.7 cm^3^) while the median mitotic count was 16 (range 3–42). The small sample size precluded meaningful comparison to the 41 dogs without metastasis. Dogs with narrow (*N* = 6) or complete (*N* = 25) margins had improved outcomes compared to dogs with incomplete margins (*N* = 14) (Figure 2C and Table 2, *p* = 0.0024). The presence of invasion (*p* = 0.25), mitotic count (*p* = 0.36), and use of systemic therapy (*p* = 0.92) were not associated with overall survival (Supplementary Figures 1A–C).

**Table 2:** Univariable analysis for potential prognostic tumor indicators in dogs with pulmonary adenocarcinoma treated with surgery with or without adjuvant systemic therapy.

Tumor variable	Group stratification based on median value	Median survival time (days)	*p*-value
Tumor volume	≤29.5	541	**0.0026**
	>29.5	219	
Mitotic count	≤9	318	0.36
	>9	339	
DARPP_N_	≤0.15	407	0.059
	>0.15	206	
DARPP_C_	≤0.125	407	0.12
	>0.125	219	
EGFR	≤1.26	339	0.39
	>1.26	315	
VEGFR2	≤1.5	419	**0.042**
	>1.5	276	

Statistically significant (*p* < 0.05) values are outlined in bold.

### 3.2 Dopamine signaling protein expression

**Figure 3:**
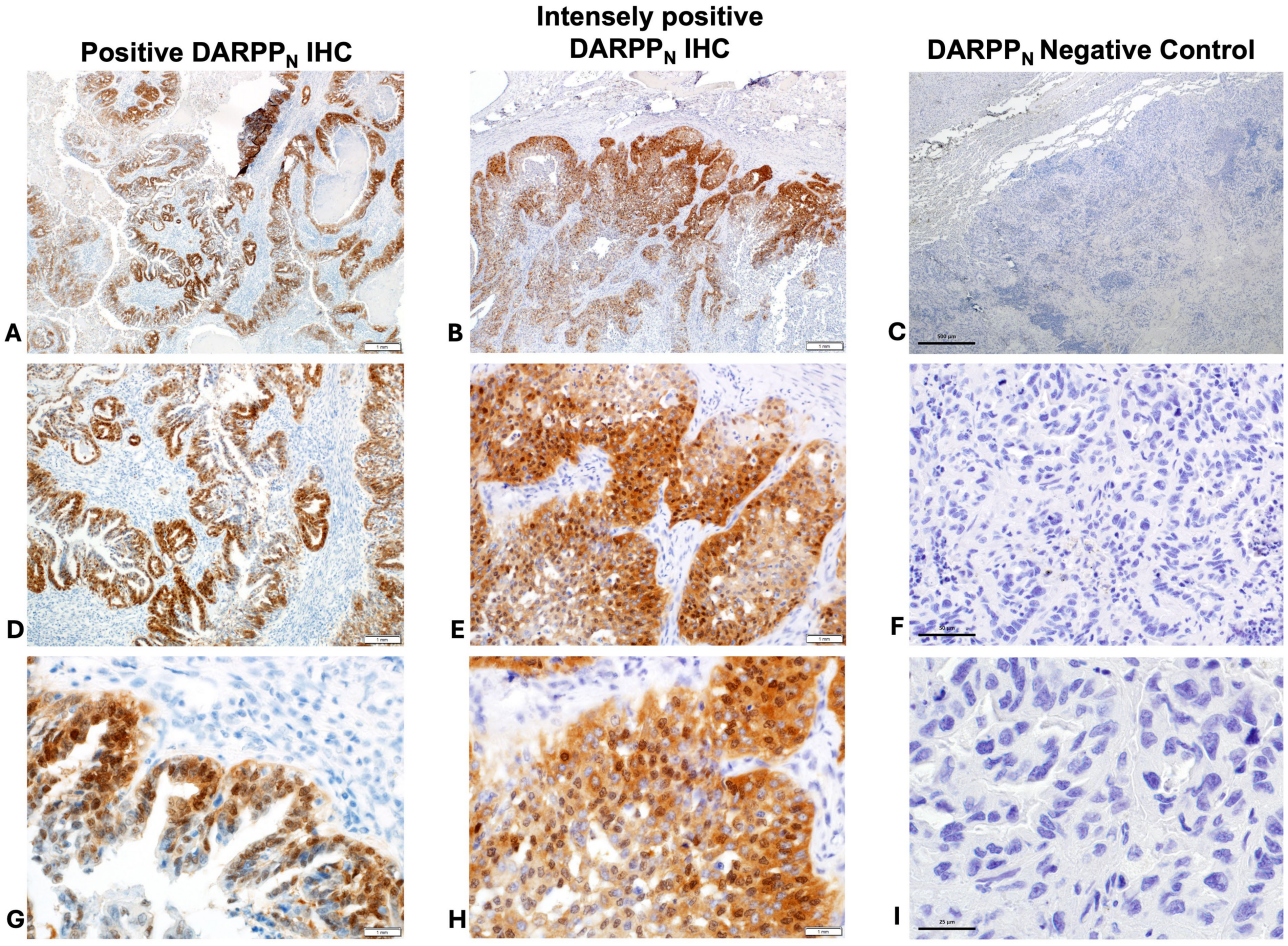
Positive immunohistochemistry (IHC) staining for DARPP_N_ (**A** = 40x magnification, **D** = 200x magnification, **G** = 400x magnification); intensely positive staining for DARPP_N_ (**B** = 40x magnification, **E** = 200x magnification, **H** = 400x magnification), and DARPP_N_ negative control (**C** = 40x magnification, **F** = 200x magnification, **J** = 400x magnification).

**Figure 4:**
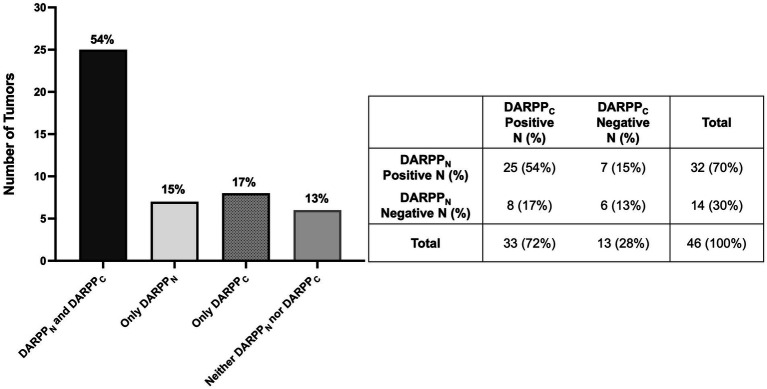
Bar graph **(A)** with associated 2 × 2 table **(B)** demonstrating the number and percentage of primary lung adenocarcinomas (*N* = 46) with positive and negative tumor cell immunostaining for both DARPP_N_ and DARPP_C_, DARPP_N_ alone, DARPP_C_ alone, or neither DARPP_N_ nor DARPP_C_.

**Figure 5:**
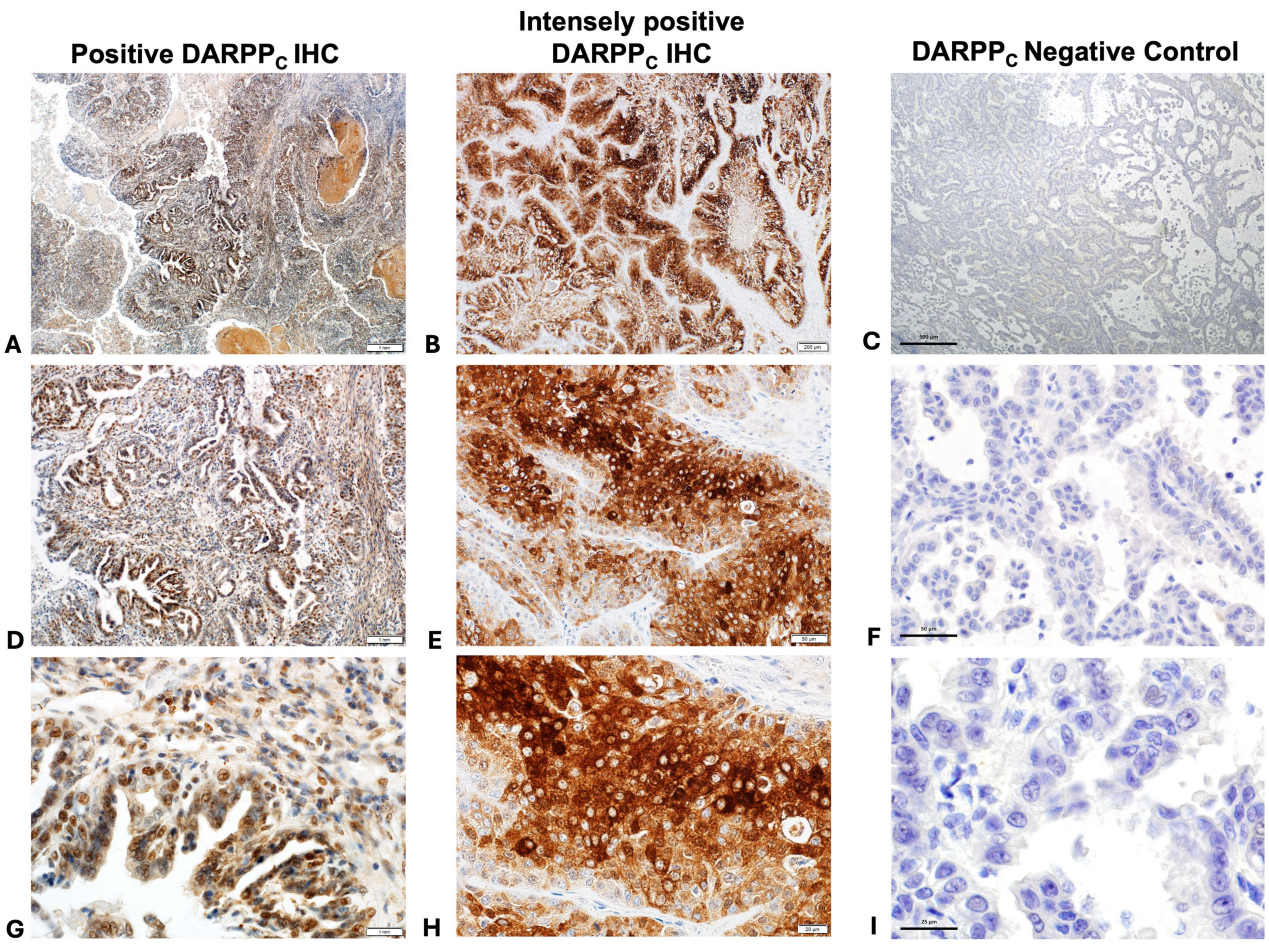
Positive immunohistochemistry (IHC) staining for DARPPC (**A** = 40x magnification, **D** = 200x magnification, **G** = 400x magnification); intensely positive staining for DARPPC (**B** = 40x magnification, **E** = 200x magnification, **H** = 400x magnification), and DARPPC negative control (**C** = 40x magnification, **F** = 200x magnification, **J** = 400x magnification).

Thirty-two (70%) tumors had expression of DARPP_N_ (i.e., the N-tersminal of DARPP-32) (Figures 3, 4) and 33 tumors (72%) showed expression of DARPP_C_ (i.e., the C-terminal of DARPP-32; Figures 4, 5). There was variable expression of DARPP_N_ and DARPP_C_, although 25 tumors (54%) had expression of both DARPP_N_ and DARPP_C_ isoforms (Figure 4). Six tumors (13.0%) were negative for both isoforms. Intensely positive staining for DARPP_N_ was identified in at least 20% of cells in seven tumors (15%) (Figures 3B, 3E, 3H); intensely positive staining for DARPP_C_ was identified in one tumor (2%) (Figures 5B, 5E, 5H). D2R showed negligible positivity in tumor-associated vascular endothelial cells from all tumors, and quantification was considered 0% for all samples.

### 3.3 Expression of EGFR and VEGFR2

**Figure 6:**
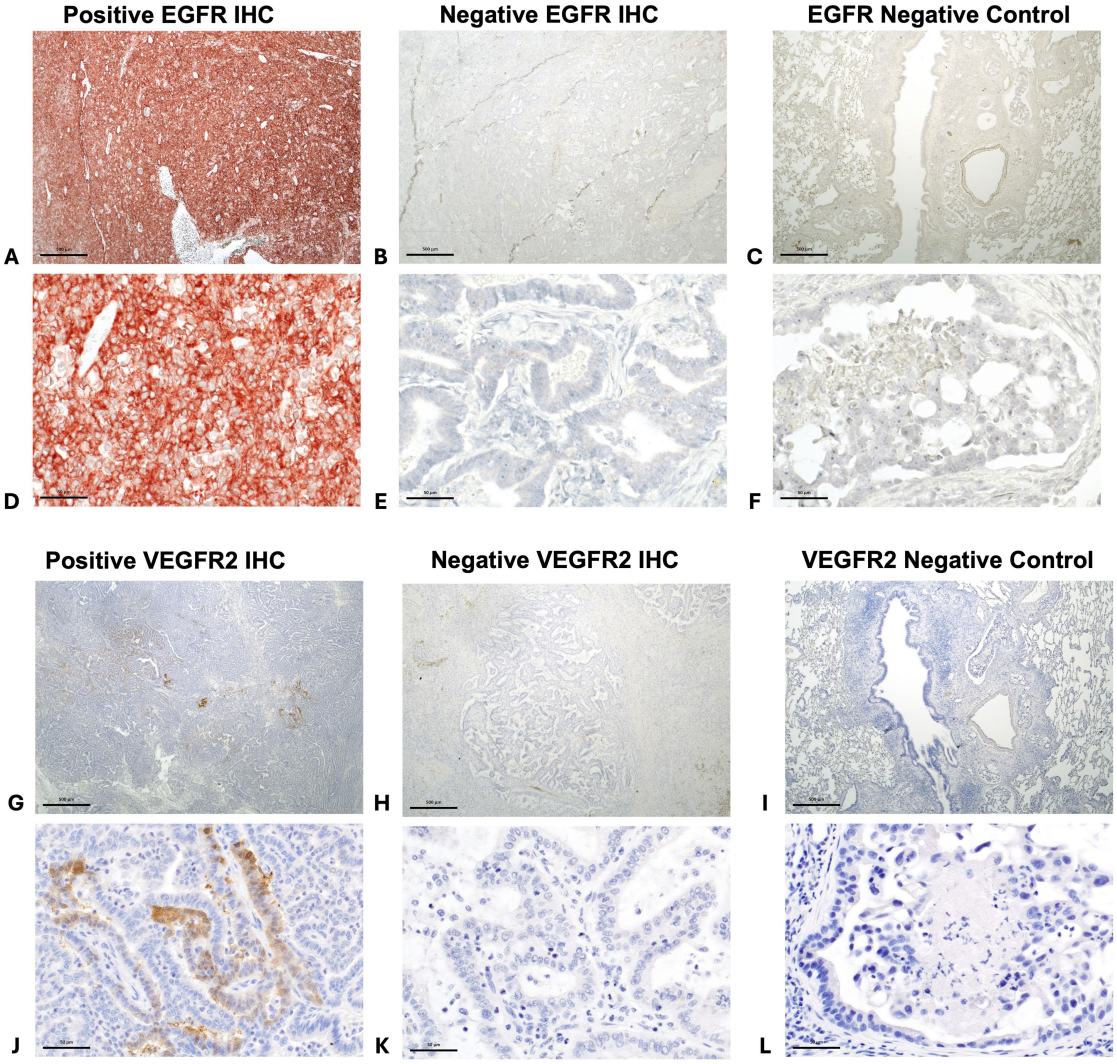
Positive immunohistochemistry (IHC) staining for EGFR in a canine pulmonary adenocarcinoma (**A** = 20x magnification and **D** = 200x magnification), negative staining for EGFR in a different tumor (**B** = 20x magnification and **E** = 200x magnification), and EGFR negative control (**C** = 20x magnification and **F** = 200x magnification). Positive intratumoral vessel staining for VEGFR2 in canine pulmonary adenocarcinomas. (**G** = 20x magnification and **J** = 200x magnification), negative staining for VEGFR2 in a different tumor (**H** = 20x magnification and **K** = 200x magnification), and VEGFR2 negative control (**I** = 20x magnification and **L** = 200x magnification).

**Figure 7:**
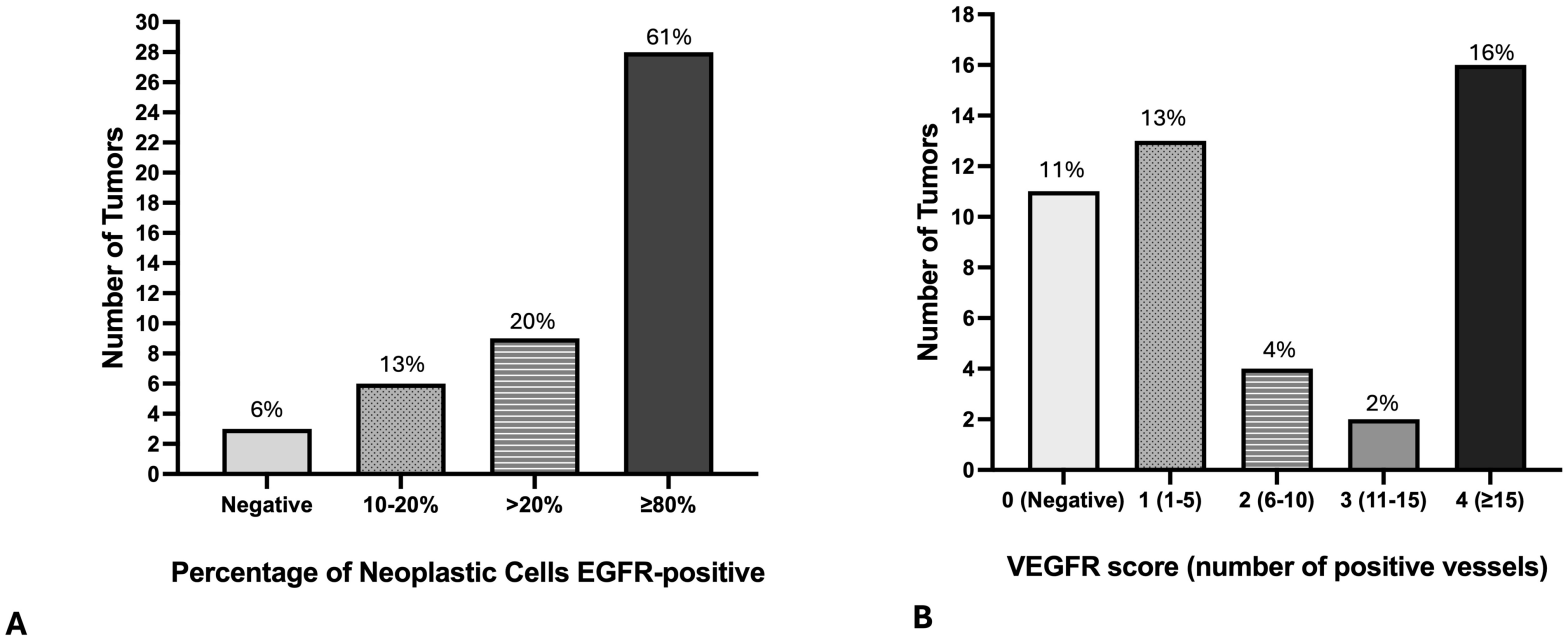
Bar graphs demonstrate the number of primary lung adenocarcinomas (*N* = 46) with the percentage of tumor cells positive for EGFR **(A)**, and with the number of intratumoral vessels with positive VGFR immunostaining **(B)**.

Forty-three (94%) tumors expressed EGFR, with only three tumors negative for EGFR (Figures 6A–F, 7A. The majority of tumors (*N* = 28; 61%) had EGFR positivity in at least 80% of neoplastic cells. Additionally, most tumors (*N* = 35; 76%) had intratumoral vessel VEGFR2 expression while 11 tumors (24%) were negative for intratumoral VEGFR2 expression (Figures 6G–L, 7B).

### 3.4 Associations between expression of target proteins

Several weak to moderate positive correlations were noted between protein IHC expression and clinicopathologic variables (Table 3). DARPP_N_ tumor expression was positively correlated to tumor volume, intratumoral VEGFR2 expression, and mitotic count. DARPP_C_ tumor expression was correlated to EGFR tumor cell expression. In addition to DARPP_N_ expression, intratumoral VEGFR2 expression was also correlated to EGFR tumor cell expression and mitotic count. DARPP_N_ and DARPP_C_ were weakly correlated to each other (*r*_s_ = 0.27), but this did not reach statistical significance (*p* = 0.07). Similarly, DARPP_N_ was weakly correlated to EGFR (*r*_s_ = 0.27), but this was not significant (*p* = 0.07). Because tumor volume has previously been correlated to tumor invasion (12), we also evaluated if the presence of absence of tumor invasion on histopathology was associated with target proteins. The presence of tumor invasion was not associated with DARPP_N_ IR (*p* = 0.26), DARPP_C_ IR (*p* = 0.57), EGFR IR (*p* = 0.56), or intratumoral vessel VEGFR2 IR score (*p* = 0.46).

**Table 3:** Clinicopathologic features correlated with DARPP32 isoforms DARPP_N_ and DARPP_C_, EGFR, and intra-tumoral VEGFR2 immunohistochemistry in canine primary pulmonary adenocarcinomas.

Correlated features		*r*_s_	*p*-value
DARPP_N_	Tumor volume	0.33	**0.027**
	Mitotic count	0.37	**0.012**
	DARPP_C_	0.27	0.073
	VEGFR2	0.34	**0.020**
	EGFR	0.27	0.070
DARPP_C_	Tumor volume	0.24	0.11
	Mitotic count	0.24	0.11
	VEGFR2	0.17	0.26
	EGFR	0.34	**0.019**
VEGFR2	Tumor volume	0.21	0.17
	Mitotic count	−0.16	0.29
	EGFR	0.42	**0.0037**
EGFR	Tumor volume	0.21	0.17
	Mitotic count	0.23	0.13
Mitotic count	Tumor volume	0.17	0.26

Statistically significant (*p* < 0.05) values are outlined in bold.

### 3.5 Associations between protein expression and outcome

**Figure 8:**
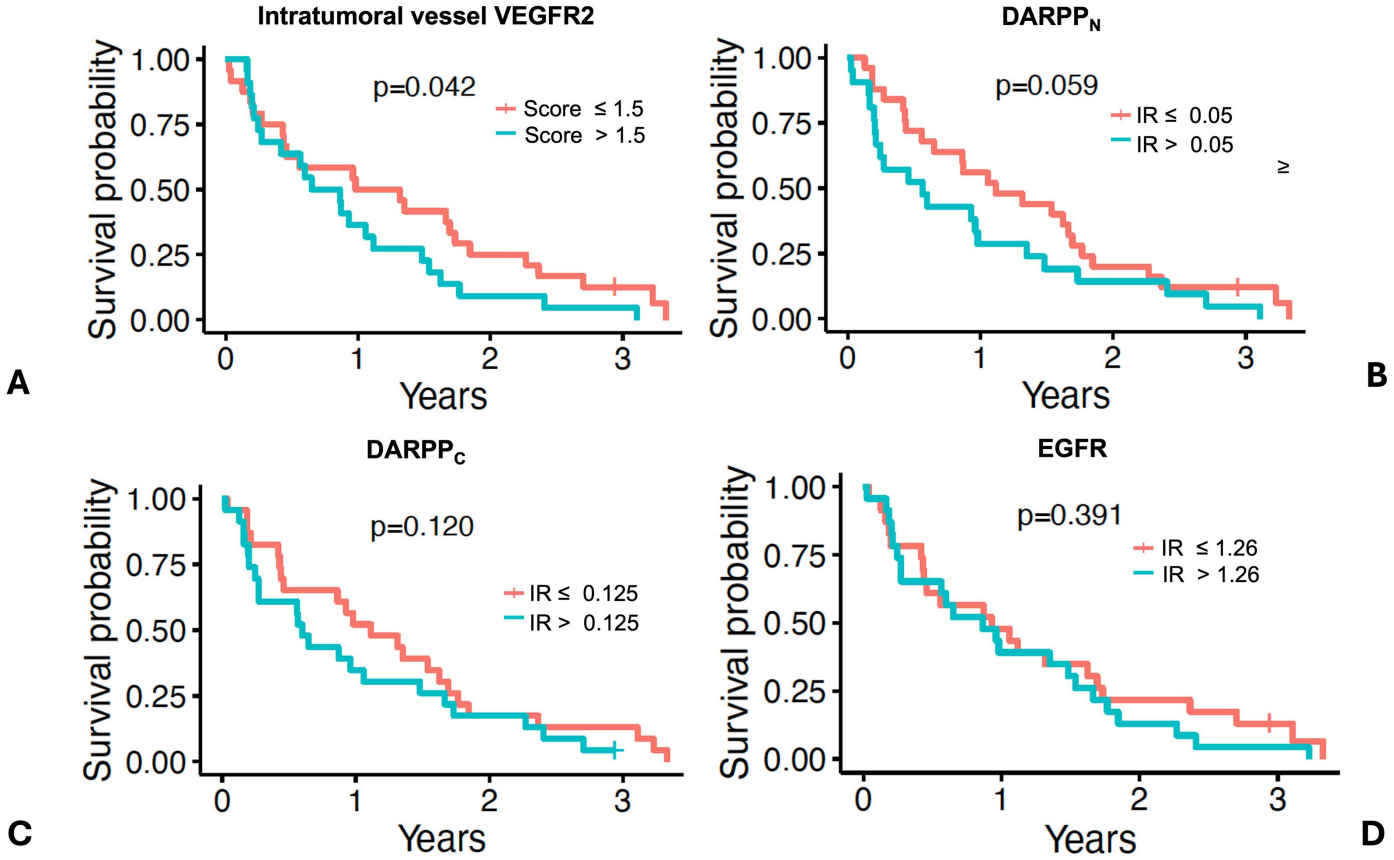
Survival in 46 dogs with primary lung adenocarcinoma following surgery, stratified by dogs above or below the median values for: **(A)** intratumoral vessel VEGFR2 score; **(B)** DARPP_N_ immunoreactivity (IR) score, **(C)** DARPP_C_ immunoreactivity (IR) score, and **(D)** EGFR immunoreactivity (IR) score. The tick mark represents one dog censored from analysis that was free of pulmonary neoplasia at the time of last follow-up. Differences in outcome were evaluated by O’Brien’s non-parametric test for numeric variables.

Dogs with intratumoral vessel VEGFR2 expression greater than the median IR score of 1.5 had a shorter MST (276 days) compared to dogs with lower intratumoral vessel VEGFR2 expression (MST 419 days) (Figure 8A and Table 2, *p* = 0.042) Dogs with DARPP_N_ expression greater than the median IR score of 0.15 had a shorter MST (206 days) compared to dogs with DARPP_N_ expression less than the median (MST 407 days), but this difference was not significant (Figure 8B and Table 2, *p* = 0.059). Similarly, dogs with DARPP_C_ expression greater than the median IR score of 0.125 had a shorter MST (219 days) compared to dogs with DARPP_C_ expression less than the median (MST 407 days), but this did not reach significance (Figure 8C and Table 2, *p* = 0.12). EGFR expression above or below the median IR score of 1.26 did not influence survival (MST 315 days versus MST 339 days, respectively, *p* = 0.391) (Figure 8D and Table 2). The small sample sizes for univariable features precluded meaningful multiple comparisons for survival.

## 4 Discussion

This study is the first to demonstrate that the majority of canine lung adenocarcinomas express DARPP-32 isoforms, similar to human lung adenocarcinomas. Data support that there are associations between DARPP-32 isoforms and clinicopathological features, including markers of cellular proliferation and angiogenesis. Additionally, DARPP_N_ tumor expression was positively correlated with tumor volume, a well described independent negative prognostic factor (10, 12, 25), and it was negatively correlated to survival.

Nearly 90% of lung adenocarcinomas evaluated stained positively for one of the DARPP-32 isoforms, but only 54% of tumors were positive for both isoforms. This finding differs from recent work in human lung adenocarcinomas, where both isoforms are generally present (1, 27). DARPP_C_, the C-terminal isoform, differs from DARPP_N_ in its ability to recognize both the full-length DARPP-32 protein and its N-terminal truncated isoform t-DARPP. By contrast, DARPP_N_ only recognizes full-length DARPP-32 by its intact N-terminal domain because the t-DARPP isoform lacks the first 36 amino acids exclusively present in the full-length DARPP-32 protein. t-DARPP, unfortunately, cannot be directly assessed by IHC. We initially aimed to perform differential IHC to calculate t-DARPP expression, as t-DARPP has been implicated in promoting cell survival, migration and is associated with poorer outcome in human NSCLC (1, 2). By subtracting the DARPP_N_ IR score from the DARPP_C_ IR score, relative expression of t-DARPP can be quantified (1, 27). While immunoreactivity against DARPP_N_ and DARPP_C_ was detected in the majority of tumors, in almost half of the tumors assessed, the DARPP_C_ IR score was lower than that of DARPP_N_. This is opposite to staining patterns described in humans (1, 27). This was unexpected and precluded us from performing differential IHC. Both antibodies were validated using canine tissue; however, it is possible that the DARPP_C_ antibody utilized has less specificity for DARPP-32 and t-DARPP than in humans. It is also possible that the age of the archived samples played a role, and that fresh tissue blocks may have provided different results. However, strong evidence supports that antigen retrieval in formalin-fixed, paraffin-embedded tissue blocks stored for several decades can be used with confidence, and that IHC staining is similar between tissue blocks and fresh frozen tissue for cytoplasmic proteins (36, 37), such as DARPP-32. Future work that incorporates gene expression analysis and gene silencing assays would be necessary to confirm and better elucidate the role of DARPP-32 and t-DARPP in canine pulmonary adenocarcinoma progression.

Surprisingly, D2R was not detected in tumor vascular endothelium in the canine pulmonary adenocarcinomas included here. Although the antibody used was validated in canine brain tissue, it is possible that prolonged storage of the paraffin-embedded tumor samples, the tumor sections selected for staining, or tissue-specific differences in antibody recognition could have contributed to this negative result. Alternatively, increased endothelial D2R expression in human lung cancer has been associated with a smoking history (26). It is possible that D2R was not detected because a link between canine primary lung tumors and second-hand smoke has not been clearly established (16). Further work is needed verify this finding, as it lends additional support for use of canine primary lung adenocarcinoma as a spontaneous tumor model of never-smoker NSCLC.

DARPP_C_ tumor protein expression was significantly positively correlated to EGFR expression, although the strength of the correlation was not high. EGFR was evaluated in this study as an indicator of cellular proliferation and survival. EGFR is a receptor tyrosine kinase that regulates these key components of tumorigenesis. EGFR overexpression has been well-documented in NSCLC and is associated with a poor prognosis (2, 19, 21–23). EGFR has also emerged as a critical drug target in treatment of EGFR mutation-positive NSCLC (2, 19, 21–23). Despite success with anti-EGFR therapies, DARPP-32 may be a pivotal player in promoting therapeutic resistance, thus stimulating the need for further investigation of its signaling pathways (2). Despite the established role of EGFR as an oncogenic driver and therapeutic target in human NSCLC, it has been sparsely evaluated in the dog. One study found that 92% of canine pulmonary carcinomas had positive EGFR protein expression, which is in line with the findings of the present study (93%), where only three tumors did not have positive staining (14). Another study reported that 73% of canine lung carcinomas were immunohistochemically positive for EGFR, using a minimum cutoff of 10% of tumor cells positive within the tissues evaluated (8). While shorter survival was observed in EGFR-positive cases in that cohort, it was not significantly different compared to dogs with EGFR-negative tumors (8). Data in this study mirror this finding, in that 80% of canine lung adenocarcinomas were EGFR-positive by IHC with greater than 20% of neoplastic cells staining positive. However, EGFR was not associated with survival following surgery. It is possible that EGFR protein signaling may have a different prognostic role in the setting of advanced, unresectable lung adenocarcinoma in the dog, or that there were too few tumors that were EGFR negative in this study, resulting in a type II error.

Tumor cell EGFR expression in canine lung adenocarcinomas was also positively correlated to intratumoral vessel VEGFR2 expression. In human NSCLC, VEGFR2 protein expression within neoplastic cells has been associated with reduced progression-free and overall survival (38). In canine lung adenocarcinoma, VEGFR2 protein expression was identified by IHC in 50% of tumors evaluated (14). However, in that study, the percentage of VEGFR2 positive cells was higher in normal lung tissue than tumor tissue, raising the question of VEGFR2 significance in this tumor type (14). Due to the well-established role that VEGFR2 plays in tumor angiogenesis, we studied intratumoral endothelial cell VEGFR2 protein expression (39). DARPP_N_ was significantly positively correlated to intratumoral vessel VEGFR2 expression in this study. Additionally, dogs with higher intratumoral vessel VEGFR2 expression had significantly shorter survival compared to dogs with lower expression. This finding supports further investigation of intratumoral vessel VEGFR2 protein signaling in canine lung adenocarcinomas given targeted therapies are commercially available. Indeed, members of our group recently demonstrated that elevated t-DARPP isoform expression is associated with poor overall survival in human lung adenocarcinoma patients and may represent a therapeutic target to slow progression (1). Here, dogs with higher tumor DARPP_N_ or DARPP_C_ expression had shorter survival compared to dogs with lower tumor expression, although differences did not reach significance.

Tumor volume is a consistent prognostic factor for outcome in canine pulmonary adenocarcinomas (8–15). DARPP_N_ immunoreactivity was significantly positively correlated to tumor volume in this study, as well as to mitotic count, further supporting it may play a role in promoting tumor proliferation and growth.

One strength of this study was that it evaluated outcome in dogs with primary lung adenocarcinoma that achieved local control following surgery, with or without adjuvant systemic therapy. Systemic therapy was not standardized and did not improve outcome in this cohort of dogs with pulmonary adenocarcinoma, consistent with prior literature (11–13, 18, 25). However, it is possible that DARPP-32 proteins may impact therapeutic response and standardized adjuvant therapy could improve efforts to evaluate this potential. Despite validation of DARPP-32 antibodies in canine tissue, it is also possible that selection and validation of additional antibodies may have altered our ability to detect t-DARPP. While the inclusion of cases confirmed by second opinion histopathology strengthens the findings of this study, it may have also contributed to a degree of selection bias. The same pathologist performed the second opinion histopathology and final review of the IHC scores; the second opinion histopathology was completed 2.5 years prior to IHC staining and subsequent analysis. Finally, the relatively small sample size in this study may have limited the strength of our findings.

Collectively, our findings demonstrate that DARPP-32 isoforms are expressed in most canine pulmonary adenocarcinomas, and they are correlated with tumor volume and markers of proliferation and angiogenesis. This study is the first to demonstrate a potential prognostic impact of intratumoral vessel VEGFR2 expression in dogs with primary pulmonary adenocarcinoma. These preliminary results support further investigation of DARPP-32 and intratumoral VEGFR2 protein signaling in studies evaluating natural animal models of never-smoker NSCLC.

## Data Availability

The original contributions presented in the study are included in the article/Supplementary material, further inquiries can be directed to the corresponding author/s.
