# Supplementary material for: Immunohistochemical characterization and potential prognostic relevance of dopamine signaling in canine pulmonary adenocarcinoma
Source: Front Vet Sci. 2025 Sep 3;12:1552345. doi: 10.3389/fvets.2025.1552345 (PMC12442036; doi:10.3389/fvets.2025.1552345)

Supplemental Data

Supplemental Figure 1.

Survival in 46 dogs with primary lung adenocarcinoma following surgery, stratified by: (A) dogs with (N=34) or without (N=12) histopathologic evidence of tumor invasion to neighboring tissues; (B) dogs with mitotic count greater than or less than the median mitotic count of 9; (C) dogs that did (N=8) or did not (N=38) receive adjuvant therapy postoperatively. The tick mark represents one dog censored from analysis that was free of pulmonary neoplasia at the time of last follow-up. Differences in outcome were evaluated by log-rank test for categorical variables and O'Brien's non-parametric test for numeric variables.

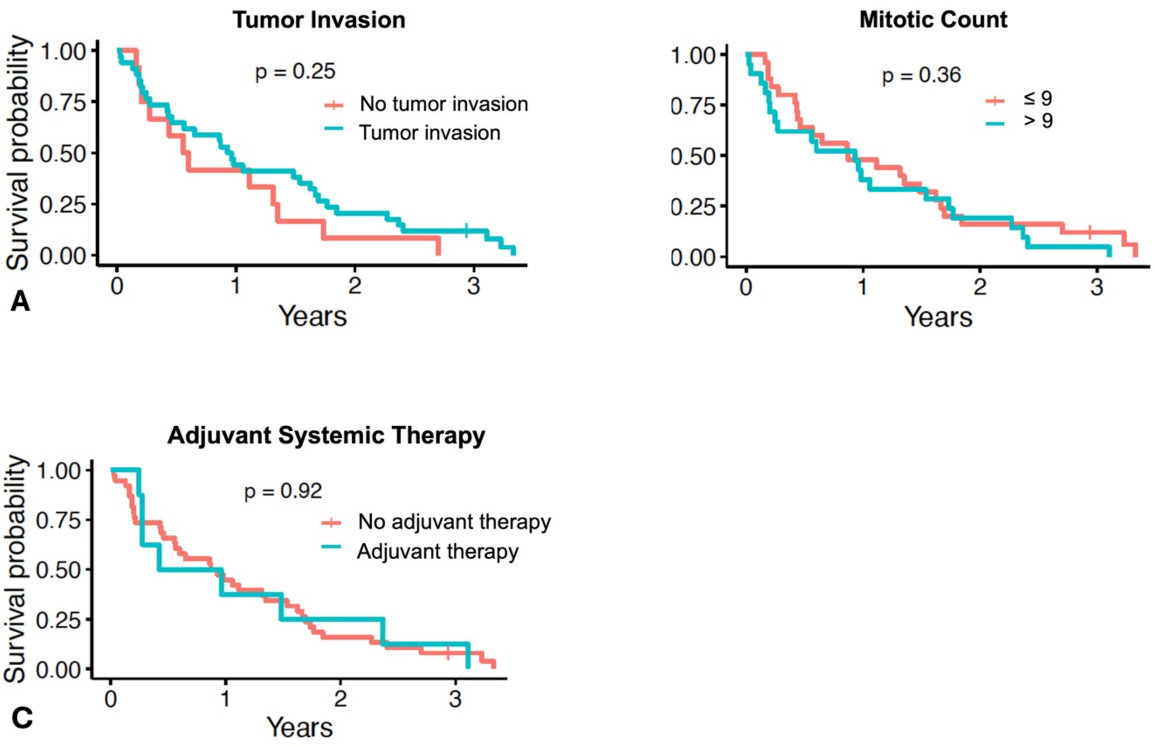

Supplement: Supplementary file 1 [file Data_Sheet_1.pdf]
